# Supplementary material for: Hot spot prediction in protein-protein interactions by an ensemble system
Source: BMC Syst Biol. 2018 Dec 31;12(Suppl 9):132. doi: 10.1186/s12918-018-0665-8 (PMC6311905; doi:10.1186/s12918-018-0665-8)
Supplement: Supplementary file 2 — All relevant MATLAB code. A simple Matlab implement of our predictor. (ZIP 19,742 kb) [file 12918_2018_665_MOESM2_ESM.zip › Additional file 2/Read ASA/ASA website/dbMPIKT/4OZGF.html]

 NetSurfP - Protein Surface Accessibility and Secondary Structure Predictions

  

|  |  |  |
| --- | --- | --- |
|  |  | NetSurfP - Protein Surface Accessibility and Secondary Structure Predictions  Technical University of Denmark |

  


---

```
# For publication of results, please cite:
# A generic method for assignment of reliability scores applied to solvent accessibility predictions.
# Bent Petersen, Thomas Nordahl Petersen, Pernille Andersen, Morten Nielsen and Claus Lundegaard
# BMC Structural Biology 2009, 9:51 doi:10.1186/1472-6807-9-51
#
# Column 1: Class assignment - B for buried or E for Exposed - Threshold: 25% exposure, but not based on RSA
# Column 2: Amino acid
# Column 3: Sequence name
# Column 4: Amino acid number
# Column 5: Relative Surface Accessibility - RSA
# Column 6: Absolute Surface Accessibility
# Column 7: Z-fit score for RSA prediction
# Column 8: Probability for Alpha-Helix
# Column 9: Probability for Beta-strand
# Column 10: Probability for Coil
E M  4OZGF                  1    0.407  81.461  -1.308   0.004   0.138   0.858
E G  4OZGF                  2    0.515  40.530   0.737   0.002   0.816   0.182
B V  4OZGF                  3    0.139  21.303  -0.336   0.001   0.900   0.099
E S  4OZGF                  4    0.455  53.303   0.294   0.002   0.816   0.182
B Q  4OZGF                  5    0.166  29.630  -0.185   0.004   0.616   0.381
E S  4OZGF                  6    0.359  42.051   0.342   0.004   0.197   0.799
E P  4OZGF                  7    0.467  66.239  -1.136   0.018   0.047   0.935
E S  4OZGF                  8    0.491  57.522  -0.747   0.056   0.142   0.802
B N  4OZGF                  9    0.261  38.181  -1.029   0.021   0.451   0.528
E K  4OZGF                 10    0.298  61.196   0.127   0.018   0.846   0.136
E V  4OZGF                 11    0.376  57.791  -0.845   0.018   0.846   0.136
E T  4OZGF                 12    0.288  40.015   0.381   0.018   0.846   0.136
E E  4OZGF                 13    0.410  71.540   0.783   0.004   0.514   0.481
E K  4OZGF                 14    0.533 109.597   0.773   0.004   0.085   0.910
E G  4OZGF                 15    0.689  54.224  -0.640   0.005   0.015   0.979
E K  4OZGF                 16    0.473  97.337   0.701   0.004   0.197   0.799
E D  4OZGF                 17    0.552  79.572   1.297   0.003   0.718   0.279
B V  4OZGF                 18    0.069  10.605   0.022   0.001   0.900   0.099
E E  4OZGF                 19    0.423  73.881   1.473   0.001   0.959   0.040
B L  4OZGF                 20    0.054   9.942   0.278   0.001   0.959   0.040
E R  4OZGF                 21    0.394  90.203   1.302   0.001   0.959   0.040
B C  4OZGF                 22    0.031   4.395  -0.084   0.001   0.900   0.099
E D  4OZGF                 23    0.445  64.182   1.514   0.001   0.900   0.099
E P  4OZGF                 24    0.299  42.456  -0.830   0.002   0.816   0.182
E I  4OZGF                 25    0.344  63.621  -0.801   0.004   0.420   0.576
E S  4OZGF                 26    0.588  68.867  -2.314   0.005   0.045   0.951
E G  4OZGF                 27    0.571  44.953  -1.734   0.005   0.015   0.979
B H  4OZGF                 28    0.277  50.386  -0.470   0.004   0.138   0.858
B T  4OZGF                 29    0.211  29.307  -0.543   0.021   0.451   0.528
B A  4OZGF                 30    0.098  10.811   0.367   0.018   0.846   0.136
B L  4OZGF                 31    0.027   4.925   0.636   0.011   0.918   0.071
B Y  4OZGF                 32    0.062  13.292   0.728   0.011   0.918   0.071
B W  4OZGF                 33    0.030   7.239   0.464   0.011   0.918   0.071
B Y  4OZGF                 34    0.054  11.476   0.965   0.011   0.918   0.071
B R  4OZGF                 35    0.132  30.159   0.745   0.018   0.846   0.136
B Q  4OZGF                 36    0.121  21.628   0.586   0.022   0.552   0.426
E S  4OZGF                 37    0.460  53.877   1.796   0.005   0.045   0.951
E L  4OZGF                 38    0.518  94.864   0.123   0.005   0.015   0.979
E G  4OZGF                 39    0.837  65.840  -1.849   0.003   0.003   0.994
E Q  4OZGF                 40    0.450  80.388   0.599   0.018   0.019   0.964
E G  4OZGF                 41    0.379  29.812  -0.144   0.018   0.088   0.893
B L  4OZGF                 42    0.102  18.621   0.716   0.005   0.336   0.660
B E  4OZGF                 43    0.269  47.047   0.559   0.002   0.816   0.182
B F  4OZGF                 44    0.093  18.725   0.403   0.001   0.959   0.040
B L  4OZGF                 45    0.023   4.175   1.070   0.001   0.959   0.040
B I  4OZGF                 46    0.026   4.828   0.197   0.000   0.983   0.017
B Y  4OZGF                 47    0.072  15.451   0.109   0.000   0.983   0.017
B F  4OZGF                 48    0.056  11.219   0.257   0.001   0.959   0.040
B Q  4OZGF                 49    0.152  27.111  -0.004   0.002   0.816   0.182
E G  4OZGF                 50    0.317  24.979  -1.021   0.005   0.336   0.660
E N  4OZGF                 51    0.551  80.696  -1.560   0.018   0.047   0.935
E S  4OZGF                 52    0.546  64.050  -1.239   0.018   0.047   0.935
E A  4OZGF                 53    0.451  49.733  -1.132   0.064   0.216   0.721
E P  4OZGF                 54    0.496  70.368  -0.719   0.022   0.359   0.619
E D  4OZGF                 55    0.443  63.851  -0.618   0.021   0.279   0.699
E K  4OZGF                 56    0.500 102.932  -0.724   0.056   0.142   0.802
E S  4OZGF                 57    0.630  73.848  -2.387   0.018   0.019   0.964
E G  4OZGF                 58    0.678  53.327  -1.731   0.018   0.019   0.964
E L  4OZGF                 59    0.303  55.498  -1.197   0.018   0.047   0.935
B P  4OZGF                 60    0.275  39.093  -0.687   0.018   0.047   0.935
E S  4OZGF                 61    0.597  69.968  -0.796   0.004   0.085   0.910
E D  4OZGF                 62    0.553  79.702  -1.077   0.004   0.138   0.858
B R  4OZGF                 63    0.279  63.799  -0.358   0.004   0.514   0.481
B F  4OZGF                 64    0.070  14.009  -0.876   0.002   0.816   0.182
E S  4OZGF                 65    0.278  32.593  -0.127   0.001   0.900   0.099
B A  4OZGF                 66    0.130  14.359  -2.419   0.002   0.816   0.182
E E  4OZGF                 67    0.406  70.841  -0.899   0.002   0.816   0.182
E R  4OZGF                 68    0.396  90.776  -0.745   0.004   0.514   0.481
E T  4OZGF                 69    0.478  66.285  -1.242   0.004   0.138   0.858
E G  4OZGF                 70    0.681  53.563  -2.133   0.005   0.045   0.951
E G  4OZGF                 71    0.652  51.312  -1.333   0.005   0.045   0.951
E S  4OZGF                 72    0.379  44.454  -0.788   0.005   0.262   0.733
E V  4OZGF                 73    0.378  58.037   0.324   0.003   0.718   0.279
B S  4OZGF                 74    0.058   6.844   0.082   0.001   0.900   0.099
B T  4OZGF                 75    0.248  34.411   0.554   0.001   0.900   0.099
B L  4OZGF                 76    0.032   5.823   0.070   0.001   0.959   0.040
E T  4OZGF                 77    0.269  37.283   0.655   0.001   0.959   0.040
B I  4OZGF                 78    0.048   8.824   0.086   0.001   0.900   0.099
E Q  4OZGF                 79    0.418  74.601   0.684   0.001   0.900   0.099
E R  4OZGF                 80    0.376  86.127  -0.964   0.003   0.718   0.279
B T  4OZGF                 81    0.246  34.176  -0.556   0.005   0.262   0.733
E Q  4OZGF                 82    0.473  84.406   0.392   0.004   0.197   0.799
E Q  4OZGF                 83    0.489  87.318  -0.590   0.004   0.197   0.799
E E  4OZGF                 84    0.604 105.589  -1.136   0.004   0.138   0.858
E D  4OZGF                 85    0.335  48.216  -0.512   0.004   0.138   0.858
B S  4OZGF                 86    0.229  26.874  -0.826   0.004   0.514   0.481
B A  4OZGF                 87    0.080   8.772  -1.501   0.002   0.816   0.182
B V  4OZGF                 88    0.148  22.809   0.611   0.001   0.900   0.099
B Y  4OZGF                 89    0.032   6.881   0.781   0.001   0.900   0.099
B L  4OZGF                 90    0.068  12.487   0.242   0.001   0.900   0.099
B C  4OZGF                 91    0.035   4.956  -0.268   0.002   0.816   0.182
B A  4OZGF                 92    0.112  12.342  -0.891   0.004   0.616   0.381
B S  4OZGF                 93    0.138  16.174  -0.512   0.004   0.420   0.576
B S  4OZGF                 94    0.295  34.586  -1.374   0.005   0.262   0.733
E F  4OZGF                 95    0.320  64.244  -1.916   0.004   0.138   0.858
E R  4OZGF                 96    0.376  85.990  -1.777   0.018   0.088   0.893
E F  4OZGF                 97    0.334  66.994  -2.181   0.018   0.047   0.935
E T  4OZGF                 98    0.359  49.793  -1.429   0.018   0.047   0.935
E D  4OZGF                 99    0.324  46.703  -1.617   0.018   0.047   0.935
E T  4OZGF                100    0.376  52.165  -1.513   0.018   0.088   0.893
E Q  4OZGF                101    0.323  57.724  -1.669   0.019   0.141   0.840
E Y  4OZGF                102    0.355  75.949  -0.831   0.019   0.141   0.840
E F  4OZGF                103    0.395  79.277  -0.915   0.020   0.205   0.775
E G  4OZGF                104    0.413  32.542  -0.896   0.020   0.205   0.775
E P  4OZGF                105    0.380  53.908  -0.854   0.019   0.141   0.840
E G  4OZGF                106    0.422  33.211  -0.807   0.020   0.205   0.775
E T  4OZGF                107    0.368  51.083  -0.657   0.021   0.279   0.699
E R  4OZGF                108    0.383  87.730  -0.525   0.022   0.359   0.619
B L  4OZGF                109    0.237  43.376  -1.500   0.021   0.451   0.528
E T  4OZGF                110    0.407  56.520  -1.242   0.022   0.552   0.426
E V  4OZGF                111    0.313  48.093  -1.753   0.021   0.451   0.528
E L  4OZGF                112    0.312  57.182  -1.241   0.020   0.205   0.775
E E  4OZGF                113    0.442  77.252  -2.056   0.018   0.088   0.893
E D  4OZGF                114    0.474  68.275  -1.509   0.018   0.088   0.893
E L  4OZGF                115    0.421  77.067  -1.921   0.019   0.141   0.840
E K  4OZGF                116    0.417  85.715  -1.713   0.019   0.141   0.840
E N  4OZGF                117    0.539  78.910  -1.732   0.018   0.088   0.893
E V  4OZGF                118    0.444  68.197  -1.576   0.018   0.088   0.893
E F  4OZGF                119    0.406  81.384  -1.287   0.018   0.088   0.893
E P  4OZGF                120    0.441  62.635  -1.417   0.019   0.141   0.840
E P  4OZGF                121    0.440  62.436  -1.085   0.020   0.205   0.775
E E  4OZGF                122    0.417  72.920  -1.233   0.022   0.359   0.619
E V  4OZGF                123    0.406  62.356  -1.640   0.021   0.451   0.528
E A  4OZGF                124    0.375  41.369  -1.045   0.022   0.359   0.619
E V  4OZGF                125    0.349  53.611  -1.882   0.022   0.359   0.619
B F  4OZGF                126    0.278  55.895  -0.995   0.021   0.279   0.699
E E  4OZGF                127    0.423  73.846  -1.522   0.018   0.088   0.893
B P  4OZGF                128    0.134  19.043  -2.404   0.018   0.047   0.935
E S  4OZGF                129    0.291  34.058  -1.450   0.018   0.088   0.893
E E  4OZGF                130    0.434  75.750  -1.683   0.020   0.205   0.775
E A  4OZGF                131    0.386  42.482  -1.979   0.020   0.205   0.775
E E  4OZGF                132    0.409  71.452  -2.304   0.021   0.279   0.699
B I  4OZGF                133    0.219  40.552  -2.282   0.020   0.205   0.775
E S  4OZGF                134    0.386  45.181  -0.606   0.004   0.085   0.910
E H  4OZGF                135    0.558 101.482  -1.071   0.018   0.047   0.935
E T  4OZGF                136    0.716  99.323  -1.375   0.005   0.015   0.979
E Q  4OZGF                137    0.595 106.267  -1.409   0.005   0.015   0.979
E K  4OZGF                138    0.469  96.453   0.622   0.005   0.262   0.733
B A  4OZGF                139    0.083   9.136  -0.050   0.002   0.816   0.182
B T  4OZGF                140    0.146  20.264   0.360   0.001   0.900   0.099
B L  4OZGF                141    0.038   6.939   0.060   0.001   0.959   0.040
B V  4OZGF                142    0.033   5.149  -0.013   0.001   0.959   0.040
B C  4OZGF                143    0.012   1.727   1.577   0.001   0.959   0.040
B L  4OZGF                144    0.088  16.076   0.420   0.001   0.959   0.040
B A  4OZGF                145    0.027   2.953   0.102   0.001   0.900   0.099
B T  4OZGF                146    0.199  27.601   0.490   0.003   0.718   0.279
E G  4OZGF                147    0.331  26.050  -0.770   0.005   0.336   0.660
B F  4OZGF                148    0.313  62.819  -1.638   0.004   0.197   0.799
E Y  4OZGF                149    0.303  64.837  -0.781   0.004   0.138   0.858
E P  4OZGF                150    0.394  55.880  -2.180   0.004   0.085   0.910
E D  4OZGF                151    0.573  82.598  -2.387   0.004   0.085   0.910
E H  4OZGF                152    0.369  67.176  -1.166   0.004   0.420   0.576
B V  4OZGF                153    0.092  14.094  -0.371   0.002   0.816   0.182
B E  4OZGF                154    0.223  38.958  -0.047   0.001   0.900   0.099
B L  4OZGF                155    0.044   8.038  -0.016   0.001   0.959   0.040
B S  4OZGF                156    0.134  15.693   0.527   0.011   0.918   0.071
B W  4OZGF                157    0.053  12.819   0.142   0.011   0.918   0.071
B W  4OZGF                158    0.256  61.616   0.434   0.001   0.900   0.099
E V  4OZGF                159    0.321  49.338   0.633   0.003   0.718   0.279
E N  4OZGF                160    0.580  84.868   0.071   0.004   0.085   0.910
E G  4OZGF                161    0.712  56.066  -1.232   0.005   0.015   0.979
E K  4OZGF                162    0.658 135.248  -0.833   0.004   0.197   0.799
E E  4OZGF                163    0.514  89.743  -1.425   0.005   0.262   0.733
E V  4OZGF                164    0.373  57.330  -1.068   0.005   0.262   0.733
E H  4OZGF                165    0.477  86.730  -1.310   0.005   0.262   0.733
E S  4OZGF                166    0.498  58.377  -1.723   0.020   0.205   0.775
E G  4OZGF                167    0.339  26.671  -1.765   0.022   0.359   0.619
B V  4OZGF                168    0.267  41.053  -1.246   0.003   0.718   0.279
B C  4OZGF                169    0.262  36.771  -0.759   0.002   0.816   0.182
E T  4OZGF                170    0.367  50.958  -0.906   0.021   0.756   0.223
B D  4OZGF                171    0.196  28.244  -0.722   0.021   0.451   0.528
E P  4OZGF                172    0.399  56.675  -1.312   0.020   0.205   0.775
E Q  4OZGF                173    0.479  85.585  -1.302   0.052   0.084   0.864
E P  4OZGF                174    0.390  55.369  -1.324   0.056   0.142   0.802
E L  4OZGF                175    0.294  53.776  -1.624   0.020   0.205   0.775
E K  4OZGF                176    0.558 114.822  -1.229   0.020   0.205   0.775
E E  4OZGF                177    0.460  80.362  -2.066   0.019   0.141   0.840
E Q  4OZGF                178    0.480  85.674  -1.284   0.019   0.141   0.840
E P  4OZGF                179    0.393  55.767  -2.515   0.020   0.205   0.775
E A  4OZGF                180    0.498  54.924  -1.799   0.021   0.279   0.699
E L  4OZGF                181    0.437  80.070  -1.192   0.021   0.279   0.699
E N  4OZGF                182    0.468  68.574  -1.654   0.019   0.141   0.840
E D  4OZGF                183    0.659  94.976  -1.928   0.005   0.045   0.951
E S  4OZGF                184    0.441  51.638  -2.493   0.004   0.085   0.910
E R  4OZGF                185    0.285  65.242  -0.005   0.004   0.514   0.481
B Y  4OZGF                186    0.125  26.606  -2.092   0.002   0.816   0.182
B A  4OZGF                187    0.114  12.563  -1.142   0.001   0.900   0.099
B L  4OZGF                188    0.101  18.530  -1.297   0.001   0.900   0.099
B S  4OZGF                189    0.105  12.341  -0.692   0.001   0.900   0.099
B S  4OZGF                190    0.059   6.973  -1.562   0.001   0.900   0.099
B R  4OZGF                191    0.176  40.304  -0.231   0.001   0.900   0.099
B L  4OZGF                192    0.037   6.701  -0.515   0.001   0.900   0.099
E R  4OZGF                193    0.288  65.906   0.744   0.002   0.816   0.182
B V  4OZGF                194    0.060   9.283   0.273   0.004   0.616   0.381
E S  4OZGF                195    0.347  40.680   0.692   0.021   0.279   0.699
E A  4OZGF                196    0.371  40.895  -0.991   0.019   0.141   0.840
E T  4OZGF                197    0.540  74.926  -0.971   0.020   0.205   0.775
E F  4OZGF                198    0.477  95.774   0.216   0.020   0.205   0.775
B W  4OZGF                199    0.180  43.314  -1.493   0.020   0.205   0.775
E Q  4OZGF                200    0.562 100.320  -0.636   0.019   0.141   0.840
E N  4OZGF                201    0.500  73.185   0.467   0.005   0.045   0.951
E P  4OZGF                202    0.465  65.969  -1.023   0.005   0.015   0.979
E R  4OZGF                203    0.645 147.591  -0.026   0.005   0.045   0.951
E N  4OZGF                204    0.370  54.227   0.848   0.004   0.420   0.576
E H  4OZGF                205    0.298  54.279   1.608   0.001   0.900   0.099
B F  4OZGF                206    0.066  13.146   1.101   0.001   0.959   0.040
B R  4OZGF                207    0.237  54.181   1.790   0.001   0.959   0.040
B C  4OZGF                208    0.023   3.159   0.957   0.001   0.959   0.040
B Q  4OZGF                209    0.234  41.739   1.291   0.001   0.959   0.040
B V  4OZGF                210    0.083  12.757   0.628   0.001   0.959   0.040
E Q  4OZGF                211    0.446  79.566   0.722   0.001   0.959   0.040
B F  4OZGF                212    0.144  28.921   0.453   0.002   0.816   0.182
E Y  4OZGF                213    0.583 124.608  -0.241   0.004   0.197   0.799
E G  4OZGF                214    0.657  51.706  -1.709   0.005   0.045   0.951
E L  4OZGF                215    0.492  90.012  -1.046   0.004   0.085   0.910
E S  4OZGF                216    0.468  54.838  -1.297   0.004   0.138   0.858
E E  4OZGF                217    0.552  96.452   0.111   0.005   0.336   0.660
E N  4OZGF                218    0.551  80.681   0.269   0.004   0.616   0.381
E D  4OZGF                219    0.262  37.797   0.373   0.002   0.816   0.182
E E  4OZGF                220    0.614 107.283   0.438   0.001   0.900   0.099
E W  4OZGF                221    0.231  55.580   1.302   0.001   0.900   0.099
E T  4OZGF                222    0.532  73.733   0.162   0.002   0.816   0.182
B Q  4OZGF                223    0.241  43.078   0.437   0.023   0.655   0.322
E D  4OZGF                224    0.597  86.013  -0.455   0.022   0.359   0.619
E R  4OZGF                225    0.498 113.927  -0.608   0.018   0.088   0.893
E A  4OZGF                226    0.553  60.996  -1.409   0.018   0.047   0.935
E K  4OZGF                227    0.627 128.933   0.064   0.005   0.045   0.951
E P  4OZGF                228    0.346  49.041  -1.254   0.004   0.085   0.910
E V  4OZGF                229    0.473  72.746  -0.492   0.004   0.138   0.858
E T  4OZGF                230    0.468  64.870  -0.725   0.004   0.138   0.858
E Q  4OZGF                231    0.400  71.511   0.112   0.004   0.197   0.799
E I  4OZGF                232    0.515  95.201  -0.822   0.005   0.336   0.660
B V  4OZGF                233    0.171  26.298  -0.060   0.004   0.514   0.481
E S  4OZGF                234    0.334  39.180  -0.128   0.023   0.655   0.322
B A  4OZGF                235    0.262  28.828   0.300   0.023   0.655   0.322
E E  4OZGF                236    0.370  64.622  -0.145   0.018   0.846   0.136
B A  4OZGF                237    0.159  17.566  -0.770   0.021   0.756   0.223
B W  4OZGF                238    0.216  51.828   0.347   0.023   0.655   0.322
E G  4OZGF                239    0.317  24.956  -2.535   0.021   0.451   0.528
E R  4OZGF                240    0.451 103.348  -0.890   0.020   0.205   0.775
E A  4OZGF                241    0.392  43.198  -2.933   0.018   0.088   0.893
E D  4OZGF                242    0.878 126.505  -0.548   0.003   0.003   0.994

Explain the output.  Go back.
```

---
